# Small islands as system and scale to examine the One Health concept

**DOI:** 10.1093/biosci/biag019

**Published:** 2026-04-06

**Authors:** Xavier G H Koenig, F B Vincent Florens, Pricila Iranah, Vikash Tatayah, Prakash N K Deenapanray

**Affiliations:** Sustainability and Climate Change Programme, Université des Mascareignes, Roches Brunes, Mauritius; Tropical Island Biodiversity, Ecology and Conservation Pole of Research, Department of Biosciences and Ocean Studies, Faculty of Science, University of Mauritius, Le Réduit 80837, Mauritius; Indian Ocean Islands Foundation, 5 The Country Side, Vivea Business Park, Saint Pierre 81406, Mauritius; Mauritian Wildlife Foundation, Vacoas, Mauritius; Sustainability and Climate Change Programme, Université des Mascareignes, Roches Brunes, Mauritius; ELIA—Ecological Living in Action Ltd., 74 Société La Flèche, La Gaulette, Mauritius

**Keywords:** One Health, islands, ecosystems, laboratories, equity

## Abstract

Small oceanic islands are among the last landmasses to be colonized by humans. Their fragile habitats and ecosystems, unprepared for anthropogenic pressures such as land cover changes and invasion by exotic species, have experienced catastrophic declines while growing human populations increasingly relied on imported environmental goods. This paper proposes a concept of One Health from a small island perspective. We synthesize results from a variety of islands to illustrate the socioecological dynamics that link nature and human health in an island context, while anchoring our analysis in Mauritius island. Shared dynamics among islands affecting ecosystems and human health show socioecological patterns that include the historical and current political ecology. By considering the island as a microcosm exhibiting similar dynamics taking place at the planetary level, globally relevant insights beyond strict environment-health interventions are evidenced, which place equity at the centre of this nexus. The perspectives that are provided also serve to explore and implement One Health approaches using the well-defined but underused unique characteristics and biota of islands.

It is increasingly recognized that our planet exhibits characteristics analogous to those of a living organism, manifested through life on Earth and expressed as biological diversity and the complex interactions between biotic and abiotic components. Apart from a continuous influx of solar energy, the biosphere functions, for all practical purposes, as a self-supporting closed system (Kleidon 2010, Solé and Levin 2022). From this whole-system perspective, the concept of health can meaningfully be applied at the planetary scale. One Health, when extended beyond its conventional focus on human and animal health, thus aligns with the notion of *One Planet, One Health*, which situates human well-being within the integrity and functioning of the Earth system as a whole (Pongsiri et al. 2019, Potter 2021, Gupta et al. 2024, Mago et al. 2024, Martens 2024). In simple terms, although planetary health has been framed in multiple ways, a consistent conclusion across these framings is that human health is fundamentally and inseparably linked to planetary health (Gupta et al. 2019).

Despite this recognition, dominant human–nature relationships remain largely shaped by instrumental capture of the natural world (Vatn 2019, Kommandeur et al. 2025, Priddat and Schlaudt 2025). While philosophical reasoning has long articulated arguments for the intrinsic value of nature (Palmer et al. 2014), and Indigenous knowledge systems emphasize relational and reciprocal human–environment connections (Redvers et al. 2022), these perspectives continue to exert limited influence on mainstream decision-making. Instead, prevailing worldviews are historically rooted in the subjugation of nature for human purposes, with religious and Cartesian traditions often cited as formative influences (Cobb 2021). In contemporary societies, this worldview is most clearly institutionalized in neoclassical economic theory, which prioritizes increasing material throughput and consumption as the primary pathway to human well-being and hedonic satisfaction (Daly and Farley 2011, Keen 2021, Diesendorf et al. 2024, Diesendorf 2025). Beyond its social and ethical implications, the economic system has profound consequences for planetary health through its thermodynamic effects. While Earth itself is a far-from-equilibrium system maintained by energy flows (Kleidon 2010, Solé and Levin 2022), the dominant economic model operates by accelerating entropic throughput via resource extraction, energy use, and waste production (Georgescu-Roegen 1971, Raine et al. 2006, Scharfenaker and Yang 2020). The growing risk of transgressing multiple planetary boundaries (Richardson et al. 2023) can be understood as an emergent manifestation of this systemic increase in entropy. Given the central role of human activities in reshaping Earth system processes (Biermann et al. 2016, Chakraborty and Qian 2024), efforts to halt or reverse environmental degradation necessarily engage with anthropogenic drivers. From this perspective, protecting planetary health may be viewed as an imperative for protecting and maintaining human health.

The connections between nature and human health operate through multiple, interacting pathways. For the purposes of this discussion, two strands are particularly salient within a One Health framework. The first concerns the emergence and spread of zoonotic diseases. Habitat loss, climate change, and the expansion of built environments increase contact between humans and animals, often favouring taxa that are more likely to host zoonotic pathogens (Keesing and Ostfeld 2021). The COVID-19 pandemic has provided a recent and globally consequential illustration of these dynamics (Lawler et al. 2021). Importantly, zoonotic risk is not simply a function of human–animal proximity but of broader ecological restructuring driven by land-use change (LUC) and biodiversity loss.

The second strand relates to human well-being. The Millennium Ecosystem Assessment (MEA 2005) has provided the conceptual framework linking ecosystem health and human well-being through a number of ecosystem functions and services (e.g., provisioning, regulating, cultural, aesthetic, spiritual) (e.g., Summers et al. 2012, Kosanic and Petzold 2020, Hernández‐Blanco et al. 2022, Zhou et al. 2025). Although initial research was constrained by methodological and framing issues (Raudsepp-Hearne et al. 2010, Duraiappah 2011, Bennett et al. 2015), subsequent research has identified socio psychological interdependencies between ecosystem health (via ecosystem services) and human well-being (Summers et al. 2012, Leviston et al. 2018, Zhou et al. 2025), as well as intersections with landscape (Wu 2013) and land use (Liu et al. 2022) changes and invasive alien species (Pejchar and Mooney 2009). Other studies have documented evidence of the impacts of biodiversity on physical health (e.g., Lovell et al. 2014, Romanelli et al. 2014, Aerts et al. 2018) and mental health (for example, Lovell et al. 2014, Korpela et al. 2018, Marselle et al. 2019). When it comes to social well-being, studies evidence positive impact on local communities of biodiversity protection on the basis of sustaining autonomy of resource rights, ways of life, and connections to nature, and this compared to communities living in areas with monocultures, degraded landscapes, or highly urbanized areas where poverty and marginality are more prevalent (Rozzi 2003). Taken together, this evidence supports the view that biodiversity conservation and ecosystem restoration are not only environmental objectives, but also strategies for improving human health and well-being (Kilpatrick et al. 2017). Accordingly, biodiversity has become increasingly central to conceptualizations of planetary health (Cena et al. 2024, Gupta 2024, Martens 2024). These interconnections are explicitly acknowledged in global policy frameworks; the Kunming–Montreal Global Biodiversity Framework, for example, articulates a 2050 vision in which biodiversity is “valued, conserved, restored and wisely used, maintaining ecosystem services, sustaining a healthy planet and delivering benefits essential for all people” (CBD 2022). This framing reflects a holistic understanding of biodiversity, health, and well-being that is closely aligned with the One Health approach.

Within this broader context, islands provide a particularly informative setting for examining the environment–health nexus. Analogous to Earth as an “island in space,” islands are bounded systems with relatively clear ecological and social boundaries, albeit open to external flows of people, goods, and species. Many oceanic islands historically supported high levels of endemic biodiversity, yet experienced substantial biodiversity loss following human colonization (Myers et al. 2000, Russell and Kueffer 2019, Fernández-Palacios et al. 2021), with rapid deforestation, species introductions, wetland drainage, and coastal modification reshaping landscapes and seascapes (Grove 1995, Crosby 2004). These trajectories have rendered islands prominent case studies for understanding the consequences of human-driven environmental change. As a result, islands have increasingly been conceptualized as natural laboratories for developing and testing approaches to biodiversity conservation, restoration, and sustainable development (Fernández-Palacios et al. 2021, Ortiz et al. 2023). From a One Health perspective, the convergence of humans, domestic and wild animals, and terrestrial, freshwater, and marine ecosystems within limited spatial extents creates distinctive opportunities for interdisciplinary research and intervention. Fountain-Jones and colleagues (2024) argue that this spatial and ecological convergence enables islands to function as models for advancing global One Health research, building on earlier propositions of islands as tractable systems for studying socioecological dynamics (Warren et al. 2015, Whittaker et al. 2017, Gugganig and Klimburg-Witjes 2021).

Despite this growing conceptualization of islands as models, important gaps remain in understanding the complex dynamics underpinning the environment–health nexus. Island systems are highly diverse, encompassing continental and oceanic islands, varying degrees of isolation, and wide gradients of socioeconomic development and environmental degradation (Fountain-Jones et al. 2024). Small Island Developing States (SIDS) (UN 1994) represent a particularly salient subset of islands in this regard. Across the Caribbean, Pacific, and Indian Ocean, SIDS are characterized by ecological sensitivity and socioeconomic vulnerability (Deenapanray and Bassi 2014, Nel et al. 2021), while contemporary patterns of biodiversity loss, health inequities, and social vulnerability are deeply entangled with historical LUC and enduring socio-political structures rooted in colonization. As such, their scale and boundedness allow socioecological interactions to be observed with a clarity that is often blurred in larger systems.

This paper contributes to the emerging literature on islands as natural laboratories by examining the complex dynamics underpinning the environment–health nexus in small island contexts. Drawing on empirical evidence from a range of island cases, and using the well-studied socioecological system of Mauritius Island as an illustrative anchor, we synthesize these insights into a causal diagram that renders visible the dominant socioecological interactions at play. In particular, we focus on two interrelated levers with net detrimental impacts on ecosystems and, by extension, on human health and well-being: (i) human-induced environmental change, and (ii) historical and contemporary issues of resource access and equity. By making these dynamics explicit, the paper aims to support more integrated One Health approaches to socioecological systems and human well-being in small island settings. Boxes 1 and 2 illustrate the broader discussion through the case of Mauritius, from ecological and social perspectives, respectively.

Box 1: **Ecosystem health and degradation in Mauritius**

Mauritius is an 8.9-million-year-old volcanic island in the Mascarene archipelago and forms part of one of the world’s biodiversity hotspots (Myers et al. 2000). Permanently settled by humans relatively late (from 1715), the island’s pre-colonial ecosystems are unusually well documented through historical accounts (Cheke and Hume 2008) and palaeontological records (Hume 2011, Rijsdijk et al. 2009). As is typical of oceanic islands, Mauritius originally supported a biota characterized by low species diversity, high endemism and ecological disharmony, notably the absence of native terrestrial quadruped mammals and the dominance of birds, bats and reptiles (Baider et al. 2010, Florens 2013a).

Human colonization triggered profound, rapid and extensive ecological transformation. Within less than four centuries, native vegetation was reduced to approximately 4.4% of its original extent (Hammond et al. 2015), persisting mainly as small, isolated fragments embedded in an agricultural landscape dominated by sugarcane (Griffiths and Florens 2006). Introduced mammals such as rats, pigs and long-tailed macaques became invasive and devastated native species that had evolved in the absence of mammalian predators. Overexploitation further accelerated biodiversity loss, including the extinction of both endemic giant tortoise species by the mid-19th century (Cheke and Hume 2008) and severe depletion of exploited palms (Maunder et al. 2002), one of which—*Hyophorbe amaricaulis*, is now the rarest species globally (Florens 2015). These pressures resulted in exceptionally high extinction rates (Florens 2013a), although intensive conservation efforts later prevented the loss of several species and produced globally recognized successes in species recovery (Bolam et al. 2021).

The speed, severity and extent of ecological degradation in Mauritius have been described as an extreme example of human impacts on island ecosystems, summarized by the observation that “all that shouldn’t be done to an island has been done to Mauritius” (Adams and Carwardine 2009). The island may therefore be viewed as both a microcosm of global environmental change and a warning of future trajectories elsewhere, where drivers of habitat transformation and biodiversity loss increasingly resemble those long present in Mauritius (Seetah et al. 2022). This makes Mauritius a valuable laboratory for studying the drivers and consequences of environmental degradation. Ongoing habitat destruction—through infrastructure expansion, real estate development, wetland infilling and activities within protected areas—has disproportionate impacts given the very limited remaining native habitat (Florens 2013a, 2013b, Laurance et al. 2012, Koenig and Deenapanray 2024). Even protected forest fragments continue to lose biodiversity through ecological debt, fragmentation and regeneration failure, with some endemic plant species persisting as only a few aging individuals (Bissessur et al. 2017).

Human–wildlife conflict has emerged as an additional pressure, most notably involving the endemic fruit bat *Pteropus niger*, which is increasingly eating commercial fruits (Bhanda et al. 2026) as its native food resources are declining in native habitats due to alien species impacts (Seegobin et al. 2026). Government-sanctioned mass culling, justified by perceived agricultural losses, has sharply reduced bat populations despite their threatened status (Hutson and Racey 2013, Vincenot et al. 2017, Kingston et al. 2018). Evidence indicates that culling failed to improve fruit production while undermining critical ecosystem services such as seed dispersal (Florens et al. 2017, Florens and Baider 2019).

Finally, as an increasingly open system, Mauritius is highly vulnerable to biological invasions. Invasive alien plants dominate native forests, comprising nearly 80% of woody stems even within protected areas (Florens et al. 2016). These invasions suppress native plant regeneration, reproduction and community diversity, with cascading effects on insects, birds and bats (Baider and Florens 2006, Florens et al. 2010, Krivek et al. 2020). Invasive alien animals—including macaques, pigs, deer and rats—further exacerbate biodiversity loss through predation, browsing and habitat degradation (Griffiths and Florens 2006, Florens 2008, Albert et al. 2021).

Box 2: **Resource access, equity and well-being in Mauritius**

In Mauritius, extensive lowland deforestation for the establishment of plantation agriculture, particularly sugarcane, was supported by the forced labour of approximately 160,000 enslaved people (Brouard 1963, Filliot 1974) during the French colonial era (1715–1810). This period also entrenched economic disparities through the consolidation of large sugar estates owned by French settler families (Crusol 2007). Under British rule (1810–1968), slavery was abolished but replaced by indentured labour from India (Brouard 1963, Rouillard and Guého 1999). Formerly enslaved people were displaced to coastal areas deemed of low economic value, where they relied on subsistence fishing or port labour (Houbert 2009). Their descendants largely remained socioeconomically marginalized due to limited asset inheritance and persistent racial discrimination. In contrast, many descendants of Indian indentured labourers gradually accumulated land and social capital, investing in education and gaining political influence (Houbert 2009, Schnepel 2009).

Following independence in 1968, economic power remained concentrated among descendants of plantation elites (Salverda 2015), who increasingly shifted toward real estate development targeting foreign investment (Koenig and Deenapanray 2024), reflecting a vertically integrated economic structure traceable to the slavery era (Boswell 2006, Claveyrolas 2012). High-end coastal developments and gated communities have contributed to community displacement and social alienation (Wortman et al. 2016; Gooding 2015). Spatially entrenched poverty is mirrored by health inequities, with low-income communities concentrated in areas with limited services and poorer health outcomes. Tourism and real estate expansion have driven urbanization and intensified pressure on coastal (marine, intertidal) and other sensitive ecosystems (Laurance et al. 2012, Hammond et al. 2015, Anisimov et al. 2020), while coastal areas hold profound cultural, livelihood, and relational value for Mauritian communities (Abib and Appadoo 2021, Merven et al. 2023). Impacts on health and well-being became especially evident during COVID-19 lockdowns and the 2020 oil spill, when coastal communities lost access to subsistence fishing and tourism income, disproportionately affecting women (de Rosnay et al. 2021, Naggea et al. 2021).

The impact of colonialism on local biodiversity contributed to the shaping of local culture. For example, enslaved, post-emancipated people and possibly even people in indentureship developed some reliance on invasive species like *Tenrec ecaudatus* (Cheke 2010), *Macaca fascicularis* (Lawler et al. 1995), *Lepus nigricollis, Rusa timorensis*, and *Sus scrofa* as free sources of protein through foraging (Seetah et al. 2022); a food practice that is still present today with culturally significant dishes like “salmi cerf,” “cari tang,” “roti cosson marron.” Today though, deer and wild boar are (mostly) in enclosed fences with armed guards, with venison and wild boar meat fetching high prices in hotels, restaurants and the local market. In parallel, monkeys have seen an increasing “sacredness” value attached to them with tensions arising on matters of monkey hunting, and monkey export for medical research. “Tang” (tenrec) meat, on the other hand, has become a niche food culture, mostly because urbanization across the landscape has made hare and tenrec rarer and harder to hunt. Thus, the access to free sources of meat protein for the self-reliant, especially historically impoverished Creole communities, has become limited to non-existent (Boswell 2006).

In addition, harvest of native plants is banned unless with a special authorization (from the National Parks and Conservation Service, and the Fisheries Division). Over time, ongoing declines in biodiversity coupled to loss of access to native plants as a resource, leads to loss of traditional and ancestral forms of knowledge on medicinal plants and plants of food value. That cultural loss is further entrenched by the regulation of healing whereby the legal landscape erases Creole traditional medicine and knowledge while uplifting Ayurvedic and Chinese healing techniques, especially through the Ayurvedic and Other Traditional Medicines Act 1989 (Urquiza-Haas and Cloatre, 2023).

## Agriculture and early land use changes

In many SIDS, agricultural expansion has been the largest historical driver of deforestation. In the Caribbean and Indian Ocean, plantation economies based on sugar, cotton, and later tea and rubber drove extensive lowland deforestation and mangrove clearance, while in the Pacific, colonial extractive regimes centred on copra, timber, phosphate mining, and strategic military infrastructure similarly transformed island ecologies (Lewis 1999, Grove and Rackham 2001). These LUCs have disrupted Indigenous and local food systems, replacing diverse subsistence practices with export-oriented monocultures and increasing dependence on imported foods. The resulting nutritional transitions—characterized by declining dietary diversity and rising consumption of ultra-processed foods—have contributed to the high burden of non-communicable diseases now observed across SIDS (McElroy and de Albuquerque 1998, Connell 2013). From a One Health perspective, these shifts altered human–animal–environment relationships, increasing vulnerability to malnutrition, zoonotic disease exposure, and environmental health risks (Destoumieux-Garzón et al. 2018).

In terms of agricultural practices currently adopted, intensive tillage and the removal of vegetation continue to disrupt soil structure, leading to compaction and reduced water infiltration, which increases surface runoff and flash flooding (Atwell et al. 2017, Rioux et al. 2017). On the other hand, forests in some cases are still cleared for timber and food production, through techniques including slash and burn agriculture, leading to the depletion of soil organic matter and increased soil acidity (Fu et al. 2022). In St. Lucia, for example, the historical legacy of the plantation economy and more recent encroachment into marginal, steep-slope areas for banana cultivation have caused significant watershed degradation (Mycoo et al. 2017). The loss of vegetation, topsoil and other ecosystem components and functions resulting from land degradation has a negative feedback effect on agricultural production and, hence, on food security and livelihoods (e.g., Lowitt et al. 2015, Wairiu 2017, Connell et al. 2019, Atzori et al. 2024), as well as the regulation and provision of water (e.g., degradation of watersheds) (e.g., Emmanuel and Clayton 2017, Gheuens et al. 2019, Mycoo and Roopnarine, 2024). Notably, this extends to coastal livelihoods as the topsoil washed into rivers and out to sea creates siltation, which blankets coral reefs, reducing their productivity and compromising near-shore fisheries (Mercer et al. 2014, Rioux et al. 2017, De Rosnay et al. 2021).

The formal abolition of slavery in Caribbean and Indian Ocean SIDS did not dismantle the plantation systems. Instead, it was frequently replaced by indentured labour regimes, particularly involving workers from South Asia, reinforcing ethnic, class, and spatial inequalities (Northrup 1995, Allen 1999). In the Pacific, colonial administrations often displaced Indigenous communities from fertile coastal and lowland areas into more marginal environments to facilitate plantation agriculture, mining, or military use. Across regions, formerly enslaved, indentured, or Indigenous populations were disproportionately confined to environmentally exposed zones—coastal fringes, floodplains, and degraded uplands—where livelihoods relied on small-scale fishing, informal agriculture, or wage labour (Pelling and Uitto 2001, Kelman et al. 2015, Komugabe-Dixson et al. 2019). These same areas are now among the most vulnerable to sea-level rise, cyclones, flooding, and ecosystem degradation, compounding health risks and constraining adaptive capacity.

## New engines of growth: Tourism and urbanization

Following independence, development trajectories across SIDS converged around tourism, offshore finance, export processing zones, and real estate investment. In many cases, tourism is now an economic mainstay—up to two-thirds of GDP in Seychelles, for example (Thebaud et al. 2023), yet it has inflicted extensive damage on coastal ecosystems, including coral reefs and mangroves (Mercer et al. 2014, Mycoo et al. 2017). Moreover, illegal sand mining to support construction undermines beach stability, preventing self-replenishment and reducing protection against storms (Mycoo et al. 2017, Komugabe-Dixson et al. 2019). Urbanization represents a parallel and often irreversible land transformation pathway in SIDS, where built-up areas are typically of high density, with correspondingly low green areas. Across the Caribbean (Mycoo et al. 2017; Jurgens et al. 2024), Indian Ocean (Koenig et al. 2024), and Pacific islands (Campbell 2019; Komugabe-Dixson et al. 2019), urban expansion frequently converts fertile agricultural land into built environments, resulting in further biodiversity loss and declining ecosystem service provision (Fu et al. 2022, Doorga 2025). On the other hand, land reclamation and encroachment have led to the loss of up to 50% of coastal wetlands in some territories (Mycoo et al. 2017), despite being vital for water purification and as “sponges” that regulate water levels during extreme rainfall events (Rioux et al. 2017).

While these sectors have generated foreign exchange and investment, they have also reinforced highly concentrated ownership of land and capital, often benefiting political and economic elites while marginalizing local communities from decision-making and resource access (Scheyvens and Russell 2012, Fletcher et al. 2020). In the Caribbean and Indian Ocean, beachfront tourism and luxury residential developments have displaced local fishing communities and restricted public access to coastal commons, while in the Pacific, large-scale resorts and infrastructure projects have intensified pressures on fragile reef–lagoon systems (Blythe et al. 2018, Hoegh-Guldberg et al. 2018). These coastal and marine ecosystems—coral reefs, mangroves, and seagrass meadows—are central to food security, livelihoods, coastal protection, and cultural identity across SIDS (Mercer et al. 2014, Rioux et al. 2017, De Rosnay et al. 2021). Even so, they are increasingly degraded by urbanization, pollution, overexploitation, and climate change. The loss or enclosure of these ecosystems disproportionately affects women, Indigenous peoples, and low-income households who rely most directly on nearshore resources, revealing a clear equity dimension to marine biodiversity decline within a One Health framework.

Rapid urbanization and land fragmentation across SIDS have further reduced the population’s everyday contact with intact ecosystems. In many islands, remaining natural areas are increasingly degraded, inaccessible, or commodified, contributing to environmental generational amnesia (Kahn 1999) and shifting baseline syndrome (Pauly 1995). Limited opportunities to access biodiverse spaces weakens understanding of human–nature interdependencies and constrain pro-environmental behaviour, undermining reciprocal relationships essential to One Health (Aminpour et al. 2022). Although biodiversity-rich environments underpin tourism and recreation economies, benefits remain largely extractive, with limited reinvestment in ecosystem restoration or community well-being (Hughes et al. 2017). The science linking urbanization and health and well-being is still in its infancy in SIDS (Alam and Ali, 2022), yet there are indications that ecosystem services can have positive effects on well-being in urban centres (Lapointe et al. 2021).

## Invasive species, biodiversity loss, and governance impacts on human-nature interactions

LUC has caused direct changes in ecosystem extents which triggered several indirect biophysical effects. Island ecosystems, however, are also particularly threatened by invasive alien species (IAS), whether introduced voluntarily or not. Again, their small size and isolation, while being rich in endemic species, make islands especially vulnerable to biological invasions (Bodey et al. 2022). Predatory IAS such as cats, mongooses, and monkeys create intense predator-prey dynamics that lead to rapid extirpation of native birds, reptiles, and land snails, while competitive IAS (plants, insects, vertebrates) displace native species and alter community structure (Russell et al. 2017). By changing soil-nutrient cycles, reducing inshore productivity, and disrupting pollination and seed-dispersal services, IAS diminish key ecosystem functions on which island livelihoods depend (Russell et al. 2017, Bodey et al. 2022). Herbivorous invaders (e.g., feral goats, pigs) cause habitat loss, soil erosion, and altered fire regimes, physically reshaping landscapes and creating vacant niches that facilitate further invasions (Russell et al. 2017). Climate change intensifies these impacts: warming temperatures improve IAS establishment rates, and extreme events such as tropical cyclones open disturbed habitats that allow invasive plants like African tulip to expand rapidly after storms (Chan 2025). The synergistic effect of climate change and IAS therefore accelerates biodiversity loss and potential for ecosystem collapse (Chan 2025). Economically, these ecological damages translate into over US $36 billion in reported costs on islands from 1965 to 2020, prompting disproportionate management spending to curb further degradation (Bodey et al. 2022).

In the context of biodiversity governance, however, colonial legacies are evident across SIDS through the entrenchment of Western conservation paradigms that prioritize protection, enclosure, and separation of humans from nature (Neumann 2005, West et al. 2006). Protected areas—both terrestrial and marine—have expanded rapidly across the Caribbean, Pacific, and Indian Ocean, often driven by external conservation agendas linked to global biodiversity targets and donor priorities (Corson et al. 2014, Koenig et al. 2025). While these interventions can yield ecological benefits, they frequently restrict customary access to land, fisheries, and culturally significant species, disproportionately affecting Indigenous peoples and historically marginalized communities. Such exclusions undermine relational well-being and erode locally embedded stewardship systems that have historically supported sustainable human–environment interactions, a core principle of One Health (Sterling et al. 2017).

Health governance in SIDS reflects similar patterns. Colonial and post-colonial health systems have prioritized biomedical and formally codified “traditional” practices while marginalizing locally embedded healing knowledge, particularly where such practices are associated with enslaved, indentured, or Indigenous populations (Vandebroek et al. 2020). Legal restrictions on access to biodiverse landscapes and native species further accelerate the loss of ethnobotanical knowledge critical for community health, resilience, and adaptive capacity (Quave and Pieroni 2015). The erosion of biodiversity thus translates directly into cultural and health losses, reinforcing inequities across generations.

## Pollution and vector-borne diseases

Beyond the human-induced environmental changes described above, tourism and urbanization drive demand for resources and typically incur increased pollution, both chemical and biological through industrial and residential effluents, emissions, and solid waste. Environmental pollution has a direct negative impact on ecosystem and human health. For illustrative purposes, we here address water pollution specifically. With increasing water scarcity, freshwater contamination increases health risks (Parker et al. 2023). Further, marine pollution is known to have detrimental impacts on human health (e.g., Scarsi et al. 2023, Morsella et al. 2025) and food security (Chen and Stain 2026). In alignment with the use of small islands as a metaphor for the microscopic dynamics reflecting macroscopic effects in One Health discussions, Morsella and colleagues (2025) have proposed ‘a visual tool adapted from the Planetary Boundaries framework (i.e., macroscopic level) to underscore the relationship between disruptors of the marine ecosystem and the resulting health consequences for humans (i.e., microscopic level).’ Water pollution also decreases ecosystem health (Morsella et al. 2025, Sylla et al. 2025), thereby establishing the linkage between ecosystem health and human health and well-being.

Small islands are also hotspots for vector-borne and waterborne diseases that have a direct negative impact on human health and well-being. For instance, tropical islands offer the right climatic conditions (or unique ecological setting) for the emergence of arboviruses, especially under a changing climate (Mavian et al. 2018). There are also instances of the re-emergence of mosquito-borne diseases and endemicity in small island states (Teeluck et al. 2025). Under a changing climate, recent studies in the Pacific SIDS have linked human health with vector-borne and waterborne diseases (Hosking et al. 2023, Damore et al. 2026). The rising incidence of vector-borne (Nuñez Murillo et al. 2025) diseases in the Pacific SIDS has led to the launch of the Pacific vector network (Haparai et al. 2025). As discussed in the next section, climate change in small islands is compounded by environmental degradation, pointing to a potential linkage between unsustainable land use, climate change and vector-borne and waterborne diseases in SIDS.

## The compounding effects of climate change

With increasing climate change, the compounding effects of multiple climate hazards are amplified by non-climate drivers such as the unsustainable land use changes (Boodoo and Deenapanray 2021) described above. Climate change notably amplifies the increasing food insecurity and water scarcity that is due to land degradation (e.g., Guell et al. 2024, Mycoo and Roopnarine 2024) with differential impacts on vulnerable communities and the poor. One study has positively correlated the incidence of drought with markedly reduced subjective well-being, with wealth measured through an “asset score” acting as a buffer (Lohmann et al. 2019). The ripple effects of land use and landscape changes in SIDS (e.g., Hay et al. 2013, Connell 2015, Duvat et al. 2017, Mycoo et al. 2017) can be significant because of an already limited land area, island topographies and usually high population densities.

One of the most dominant linkages between climate change and health is mediated through physical loss and damage (e.g., infrastructure and natural capital), the loss of lives, the loss of livelihoods, and the psychological or mental (or secondary effects) impacts of climate change (Du Bray et al. 2017, Nunn and Kumar 2018, Lohmann et al. 2019, Furusawa et al. 2021, Kelman et al. 2021, Smith 2024, Williams 2025, Park et al. 2025). The impacts on human health can range from grief to anxiety to trauma caused by the loss of lives, loss of livelihoods, breakdown in the supply of essential services (e.g., access and mobility because of road infrastructure damage and damage to health infrastructure) and repeated exposure to climate hazards, among others. At its extreme, the psychological impact of climate change in low-lying SIDS is manifested in “climate refugees” (Lewis 2011). Also, resource extraction in small islands, although contributing to economic growth, has been shown to degrade the environment and enhance climate change, thereby resulting is loss in both ecosystem and human health (Bambrick 2018).

An emerging pathways approach to climate adaptation and resilience-building in SIDS is through better land management (e.g., Leal Filho et al. 2021, Major et al. 2021) and investments in nature-based solutions, NbS (e.g., Zari et al. 2019, Châles et al. 2023, Suedel et al. 2023, Techera 2023, Dawkins and Zhang 2025). Both measures are squarely aligned with the objectives of the UN Conventions on Combatting Desertification (UNCCD 2025) and Biological Diversity (CBD 2022) for halting and reversing land degradation, and achieving nature positive outcomes, respectively. Further, NbS has been proposed for enhancing urban resilience in several Pacific SIDS “within an urban ecosystems framework linked closely to Indigenous understandings of well-being (Kiddle et al. 2021).”

## Synthesis

This section describes the causal diagram presented in figure 1, which provides a synthesis of the complex dynamics covered in the paper. Before proceeding, however, it must be stressed that the human-induced environmental changes described herein, which result in degradation, are largely correlated with economic growth (Conrad and Cassar 2014, Seetanah et al. 2019, Tandrayen-Ragoobur and Fauzel 2021, Fauzel and Tandrayen-Ragoobur 2023). In addition to economic growth, environmental degradation in small islands is driven by financial development (Seetanah et al. 2019). In the long run, however, environmental degradation in 19 SIDS has been shown to deter economic growth (Tandrayen-Ragoobur and Fauzel 2021). These observations have been confirmed through a political ecology lens of financial maximization in the case of land use changes in Mauritius (Koenig and Deenapanray 2024), with environmental degradation being driven by poor governance (Tandrayen-Ragoobur and Fauzel 2021, Koenig and Deenapanray 2024).

**Figure 1:**
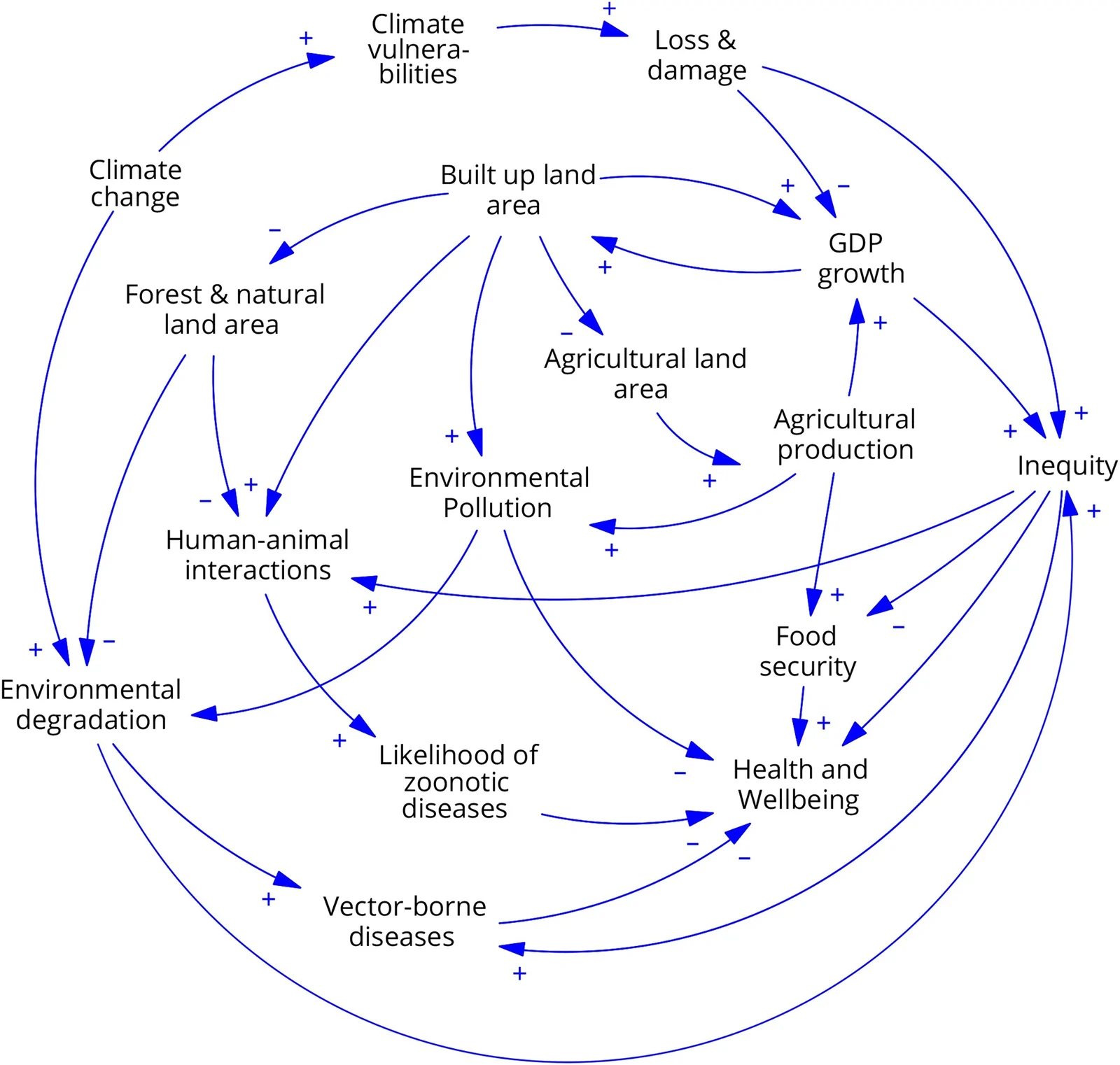
Causal diagram of primary dynamics affecting health and well-being in SIDS.

The causal diagram should be interpreted as follows. Starting with broad economic and biophysical aspects, urbanization is driving GDP growth in a self-reinforcing manner. The growth of built-up areas, in turn, translates into loss of agricultural areas, as well as forest and natural land areas, although the historical driver of deforestation has been the expansion of agriculture. Combined urbanization and loss of natural areas drive increased human-animal interactions and consequently the likelihood of emerging zoonotic diseases, affecting health and well-being. Added built-up areas also contribute to increased pollution, both chemical and biological through industrial and residential effluents, emissions, and solid waste. Agricultural production, through its inputs and interactions with soil and water resources, also contributes to environmental pollution, particularly of coastal and marine ecosystems through waterways and groundwater, which can directly affect human and animal health. Together, climate change, pollution, and the loss of natural areas are taken as drivers of environmental degradation, which is in many cases conductive for vector-borne diseases, affecting health and well-being. Health and well-being are also impacted by food security, which is tributary of the agricultural land area that is under pressure in SIDS.

Secondly, socio-political and socioeconomic issues play a clear role within the above causal chains, here captured through the lens of equity, or rather lack thereof; a pattern of inequity perpetuated by GDP growth through post-colonial systems. Since vulnerable communities are often situated in environments which are degraded and/or prone to climate risks, inequity is further exacerbated by potential loss and damage from climate change effects and/or extreme events. Their typically higher reliance on environmental resources means that they are more exposed to animal interactions, vector-borne diseases, and derive decreasing benefits from the services of degraded and degrading ecosystems, affecting food security and health. All of those, alongside mental health aspects, affect the well-being of vulnerable communities, which may be felt throughout society, and especially so through vector-borne or zoonotic diseases, should outbreaks occur.

## Conclusion

This paper has pointed to the dominant socioecological linkages evidenced across SIDS, which predominantly arise from utilitarian approaches and power dynamics, that structured access to land, to ecosystems and their services, and ultimately health and well-being. While these common linkages may reinforce the tractability of SIDS as natural laboratories to better understand, frame and research the One Health concept, all islands remain highly complex socioecosystems through their individually unique ecology, history, socioeconomic and political systems. As such, although there clearly will not be any “one size fits all” intervention for SIDS at large, the approach taken here reveals that biodiversity decline, social injustice, and health inequities are not parallel challenges but co-produced outcomes of historical and ongoing political-economic processes. A One Health approach in SIDS therefore requires more than technical integration across the environment and health, but demands explicit engagement with colonial legacies, land and resource governance, cultural recognition, and equity, recognizing that sustainable health outcomes depend on restoring both ecosystems and the social relations through which people live with them.

## Data Availability

No new data were generated or analyzed in support of this research.
